# Update on the Clinical and Molecular Characterization of Noonan Syndrome and Other RASopathies: A Retrospective Study and Systematic Review

**DOI:** 10.3390/ijms26083515

**Published:** 2025-04-09

**Authors:** Giuseppe Reynolds, Andrea Gazzin, Diana Carli, Stefania Massuras, Simona Cardaropoli, Maria Luca, Beatrice Defilippi, Marco Tartaglia, Giovanni Battista Ferrero, Alessandro Mussa

**Affiliations:** 1 Department of Public Health and Pediatrics, Postgraduate School of Pediatrics, University of Torino, 10126 Turin, Italy; giuseppe.reynolds@unito.it; 2 Department of Public Health and Pediatric Sciences, University of Torino, 10126 Turin, Italy; andrea.gazzin@unito.it (A.G.); stefania.massuras@unito.it (S.M.); simona.cardaropoli@unito.it (S.C.); beatrice.defilippi@unito.it (B.D.); 3 Clinical Pediatrics Genetics Unit, Regina Margherita Children’s Hospital, 10126 Turin, Italy; 4 Department of Medical Sciences, University of Turin, 10126 Turin, Italy; diana.carli@unito.it (D.C.); maria.luca@unito.it (M.L.); 5 Molecular Genetics and Functional Genomics, Bambino Gesù Children’s Hospital, IRCCS, 00146 Rome, Italy; marco.tartaglia@opbg.net; 6 Department of Clinical and Biological Sciences, University of Turin, 10043 Orbassano, Italy; giovannibattista.ferrero@unito.it

**Keywords:** RAS/MAPK pathway, *PTPN11*, *SOS1*, *RAF1*, congenital disorders

## Abstract

RASopathies are a diverse group of genetic conditions caused by hyperactivation of the RAS-MAPK signaling pathway, mainly inherited in an autosomal dominant manner. They present with variable features such as short stature, congenital heart defects, facial dysmorphisms, and neurodevelopmental delays. This study retrospectively analyzed 143 cases from 2003 to 2022, aiming to improve genotype–phenotype correlation knowledge for personalized care. Patients with genetically confirmed Noonan syndrome (NS) and related disorders were included, with molecular analysis performed via Sanger or parallel sequencing. Data from 906 previously reported cases were also reviewed. Among the 143 patients, most had NS (*n* = 116). *PTPN11* mutations were most frequent (61%), followed by *SOS1* (10.3%) and *RAF1* (8.6%). Cardiac anomalies were observed in 71%, with pulmonary stenosis (PS) prevalent in NS (48.3%) and hypertrophic cardiomyopathy (HCM) in NSML (40%). *PTPN11* variants were linked to PS and atrial septal defects, *SOS1* to multiple cardiopathies, and *RAF1* to HCM. Additional features included facial dysmorphisms (74.1%), short stature (62.0%), skeletal anomalies (43.1%), cryptorchidism (59.7%), and brain abnormalities (17.2%). JMML and other malignancies were seen in eight patients. This study emphasizes the importance of genotype-guided care, improved diagnosis of mild cases, and the underrecognized prevalence of neurological anomalies.

## 1. Introduction

Noonan syndrome (NS) is a clinically variable and genetically heterogeneous disorder, first described in 1968 by Jacqueline Noonan [1]. It is characterized by short stature, congenital heart disease, distinctive facial features, variable neurodevelopmental delay, and other anomalies [2,3,4]. While rare, NS is among the most common non-chromosomal disorders affecting development [4]. NS is framed in the broader group of RASopathies, conditions that are mostly transmitted as autosomal dominant traits and caused by dysregulation of the RAS-MAPK signaling pathway [4,5,6]. This family of disorders includes neurofibromatosis type 1 (NF1, OMIM 162200), cardiofaciocutaneous syndrome (CFCS, OMIM 115150), Noonan syndrome with multiple lentigines (NSML, OMIM 151100), Noonan-like syndrome with loose anagen hair or Mazzanti syndrome (NS/LAH, OMIM 607721), Costello syndrome (CS, OMIM 218040), Legius syndrome (LS, OMIM 611431), Noonan syndrome-like disorder with or without juvenile myelomonocytic leukemia (NSLL, OMIM 613563), and other clinically related conditions caused by variants in other functionally related genes [5,6,7,8,9,10].

### 1.1. Biology of the RAS/MAPK Cascade

**Figure 1:**
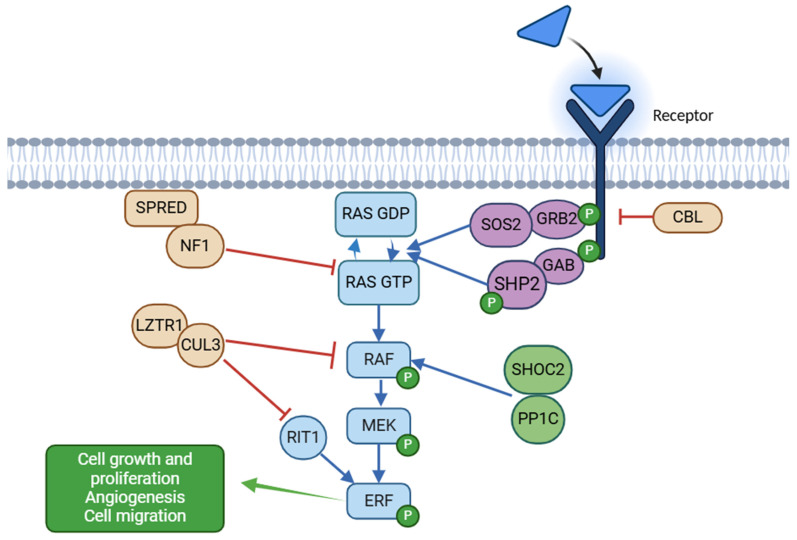
RAS-MAPK pathway and key proteins involved in its regulation. Positive modulators of signaling are divided into 3 groups according to the position in the cascade, appearing in blue, purple, and green, while negative regulators appear in orange. In RASopathies, pathway hyperactivation results from increased activity of RAS proteins, upstream regulators, and MAPK cascade components. Impaired feedback mechanisms, due to alterations in genes such as *CBL*, *NF1*, *LZTR1*, and *SPRED*, further contribute to prolonged signaling activation. “P” indicates phosphorylation.

The RAS (rat sarcoma)/MAPK (mitogen-activated protein kinase) pathway is a ubiquitous and highly conserved signal transduction cascade that enables cells to respond and adapt to extracellular stimuli such as growth factors, cytokines, and hormones, regulating proliferation, differentiation, metabolism, survival, and migration (Figure 1) [5,6]. In RASopathies, this pathway is dysregulated due to pathogenic variants in genes that either enhance RAS activity or disrupt its regulation [6]. Core components include RAS proteins encoded by *KRAS* (Kirsten rat sarcoma virus), *HRAS* (Harvey Rat sarcoma virus), and *NRAS* (neuroblastoma RAS viral oncogene homolog), which act as molecular switches cycling between inactive GDP (guanosine diphosphate)—bound and active GTP (guanosine triphosphate)—bound states. Variants such as *KRAS* p.Gly12Val or *HRAS* p.Gly12Ser promote constitutive activation and continuous downstream signaling [11,12].

Regulators such as SOS1 (Son of sevenless homolog 1), SOS2 (Son of sevenless homolog 2), SHOC2, and CBL (Casitas B-lineage Lymphoma) play essential roles in modulating RAS activity. SOS1 and SOS2, both guanine nucleotide exchange factors (GEFs), facilitate the exchange of GDP for GTP on RAS, activating it. Variants in *SOS1* increase its GEF activity and amplify RAS signaling [13]. While *SOS2* variants are less common, emerging evidence suggests that they contribute to RASopathies through similar mechanisms, albeit with distinct tissue-specific effects due to differences in expression patterns and functional interactions [14]. SHOC2, a scaffolding protein, amplifies pathway signaling by stabilizing active RAS complexes. Variants such as p.Ser2Gly modify its function, enhancing MAPK signaling in specific tissues [15]. The E3 ubiquitin ligase CBL negatively regulates the pathway by targeting RAS for degradation; *CBL* variants impair this function, prolonging RAS activation [16,17].

Downstream of RAS, the RAF (Rapidly Accelerated Fibrosarcoma) kinases (RAF1, BRAF) transmit the signal to MEK1/2, such as MAP2K1, MAP2K2 (mitogen-activated protein 2 kinases 1 and 2), and, ultimately, ERK1/2, which phosphorylate transcription factors in the nucleus to drive cell cycle progression and differentiation. Variants like *RAF1* p.Ser257Leu and *BRAF* p.Val600Glu hyperactivate this cascade [18]. Additional components, such as *PTPN11* (tyrosine-protein phosphatase non-receptor type 11) encoding SHP2 (Src homology region 2 domain-containing phosphatase-2), positively regulate the pathway by modulating receptor tyrosine kinase signaling, promoting hyperactivation [19]. *LZTR1* (Leucine-zipper-like transcriptional regulator 1), a regulator of RAS ubiquitination, plays a key role in limiting RAS activity by facilitating its degradation. Both monoallelic and biallelic inactivating variants in *LZTR1* can contribute to NS, with biallelic variants causing an autosomal recessive form, while monoallelic variants have been associated with autosomal dominant cases. Additionally, monoallelic *LZTR1* variants are implicated in schwannomatosis, highlighting the gene’s diverse, tissue-specific impact on the RAS/MAPK pathway [20,21]. Loss of LZTR1 function results in enhanced stability and activity of RAS proteins, contributing to the pathogenesis of RASopathies.

Pathogenic variants in *RIT1* (RIC-like protein without caax motif 1), a member of the RAS subfamily of small GTPases, contribute to the dysregulation of the RAS/MAPK pathway by enhancing its activation and prolonging signal transduction. These variants, such as p.Ala57Gly, increase GTP binding and reduce GTP hydrolysis, leading to constitutive pathway activation [22]. This hyperactivation affects cellular processes like proliferation and differentiation, contributing to characteristic features of RASopathies, including cardiac defects and craniofacial anomalies [23]. Overactivation of MEK1 and MEK2, deriving form variants in *MAP2K1* and *MAP2K2*, play a pivotal role in the dysregulation of the RAS/MAPK pathway by enhancing their kinase activity, which leads to aberrant phosphorylation of downstream effectors like ERK1/2 [24,25,26]. This hyperactivation disrupts cellular processes like differentiation and proliferation, underpinning the diverse phenotypic spectrum of RASopathies [5].

### 1.2. Genetic Causes of RASopathies and Genotype–Phenotype Correlations

RASopathies result from the dysregulation of the tightly controlled RAS/MAPK pathway, with variable degrees of pathway hyperactivation correlating with clinical heterogeneity [15]. Phenotypic differences in RASopathies arise from the distinct biological roles of each gene within the RAS/MAPK pathway, the degree of pathway dysregulation caused by specific variants, and the tissue-specific effects of altered signaling. For example, variants in *PTPN11*, such as p.Asn308Asp, generally result in moderate pathway hyperactivation, causing classical NS with mild to moderate developmental delays, congenital heart defects, and characteristic facial features [27]. In contrast, *KRAS* variants, such as p.Gly12Val or p.Thr58Ile, lead to severe activation and phenotypes overlapping with CFCS, including significant neurocognitive impairments [28]. Variants in *BRAF*, like p.Gln257Arg and p.Val600Glu, cause profound hyperactivation, aligning with the more severe developmental delays, craniofacial dysmorphism, and ectodermal anomalies seen in CFCS [29]. Similarly, activating variants in *HRAS*, such as p.Gly12Ser, are associated with CS, one of the most hyperactivated states among RASopathies, characterized by severe developmental delays, cardiomyopathy, and a high malignancy risk [12,30]. *SOS1* variants, typically linked to milder forms of NS, exhibit localized hyperactivation, resulting in ectodermal anomalies like curly hair and keratosis pilaris [15,31]. While *SOS2* variants are rare, they have been identified in some patients with NS phenotypes, further expanding the genotype–phenotype spectrum of RASopathies [21].

Differences in clinical severity also reflect the hierarchical positioning of proteins within the pathway; for instance, *RAF1* variants preferentially activate cardiac-specific signaling pathways, explaining the high prevalence of hypertrophic cardiomyopathy [32]. Tissue-specific expression and the timing of pathway dysregulation during development play crucial roles. For example, *SHOC2* variants uniquely amplify signaling in hair follicles and ectodermal tissues, leading to distinctive phenotypes: variants in *SHOC2*, such as p.Ser2Gly, lead to NS-LAH, a phenotype including sparse, easily pluckable hair and ectodermal anomalies due to *SHOC2*’s role as a tissue-specific MAPK scaffold [33,34]. The molecular mechanisms underlying *LZTR1* variants highlight its distinct contribution: biallelic inactivation drives global hyperactivation associated with NS, whereas monoallelic loss causes tissue-restricted phenotypes such as schwannomatosis [35,36].

The contribution of different genes to the etiology of RASopathies varies significantly, with *PTPN11* pathogenic variants being the most common, accounting for approximately 50% of Noonan syndrome cases [19,37]. *SOS1* variants follow, responsible for around 10–15% of cases, often associated with ectodermal anomalies [38]. Pathogenic variants in *RAF1* are found in about 5–10% of individuals, frequently linked to hypertrophic cardiomyopathy [39]. Recent studies have identified autosomal recessive forms of NS associated with biallelic inactivating variants of *LZTR1* and *SPRED2* (Sprouty-related, EVH1 domain-containing protein 2) [21,35,40,41]. The contribution of other known genes seems residual, with mutation frequencies from 0.5% to 2% in different studied populations [15,40,42]. To date, nearly 10% of subjects with suggestive clinical phenotypes are not molecularly solved, indicating that other genes might be implicated in these disorders or substantial clinical overlap might exist with different syndromic conditions. The prevalence and severity of clinical manifestations mirror the degree of molecular heterogeneity, and genotype–phenotype correlations have been identified at the gene and variant levels [43].

### 1.3. Objectives of the Study

Understanding the genotype–phenotype relationship in NS and related syndromes is crucial for improving diagnostic accuracy, predicting disease progression, and optimizing clinical management. Although significant progress has been made in identifying pathogenic variants associated with RASopathies, many genotype–phenotype correlations remain incompletely defined, particularly for less common genes. Additionally, patients with identical genetic variants can exhibit highly variable clinical manifestations, suggesting the influence of modifier genes, epigenetic factors, or environmental interactions.

Given the rarity and heterogeneity of RASopathies, a single-center study may be insufficient to draw robust conclusions. Therefore, to enhance the statistical power and generalizability of our findings, we combined our cohort with a pooled dataset of 906 previously reported cases. This approach allows for a more comprehensive evaluation of the variability in clinical presentation associated with specific genetic mutations. Therefore, the main objective of this retrospective study was to integrate phenotypic and genotypic data from all genetically confirmed cases of NS and related disorders diagnosed at our tertiary referral center in Northern Italy with information from cases diagnosed in other published series of patients with similar pathologies, with the aim of refining and expanding genotype–phenotype correlations in RASopathies. The insights gained from this analysis may support the development of genotype-driven clinical guidelines, facilitate early recognition of disease complications, and, ultimately, improve personalized care for individuals affected by RASopathies.

## 2. Materials and Methods

### 2.1. Study Design and Data Collection of the Retrospective Analysis of Our Cohort

Our investigation was a retrospective, observational study that included patients with a genetically confirmed diagnosis of NS and related disorders, who were diagnosed, visited, or followed up with at the Clinical Genetic Unit of the Regina Margherita Children’s Hospital of Torino between 2003 and 2022. Our center serves as the major service provider for genetic diseases in Piedmont, a large region in Northwest Italy with more than four million inhabitants. The study was conducted in accordance with the ethical principles enshrined in the Helsinki Declaration. Only patients with a molecularly confirmed clinical diagnosis and extensive clinical information were included in the study. Exclusion criteria were (a) clinical diagnosis in the absence of genetic confirmation and (b) follow-up in other centers.

### 2.2. Molecular Methods of the Retrospective Analysis of Our Cohort

From 2003 to 2011, DNA samples of patients with suspected RASopathy clinically evaluated in our Center, extracted from peripheral blood lymphocytes, were tested for variants of PTPN11, SOS1, and RAF1 using the Sanger sequencing technique. Since 2011, analyses have been performed through parallel sequencing, using custom panels designed to allow scanning of the PTPN11, SOS1, RAF1, CBL, KRAS, HRAS, BRAF, LZTR1, SOS2, RIT1, SHOC2, and MAP2K1 coding exons. Libraries were sequenced using a NextSeg550 platform (Illumina). All variants were classified according to ACMG criteria [44]. Only patients with a molecularly confirmed clinical diagnosis and extensive clinical information were included in the study. Exclusion criteria were (a) clinical diagnosis in the absence of genetic confirmation and (b) follow-up in other centers.

### 2.3. Clinical Data Collection and Phenotypic Assessment of the Retrospective Analysis of Our Cohort

For each proband, an extensive three-generation family history was systematically assessed. Recorded phenotypic data of the affected patients included clinical prenatal and postnatal features, dysmorphic facial features, presence of cardiovascular, skeletal, nervous, renal, visual, and hearing abnormalities, cryptorchidism, bleeding diathesis, cognitive impairment, and neoplasms. Anthropometric parameters, such as weight measured in kilograms with a scale and stature and head circumference measured in centimeters, respectively, with a statimeter and a tape measure, were analyzed by sex and age, and are described as standard deviation (SD).

### 2.4. Methods of the Systematic Review

The literature review was conducted on PubMed, employing keyword searches, including the following: “clinical findings” AND “Noonan syndrome” or “RASopathies”, “phenotype” AND “Noonan syndrome” or “RASopathies”, “genotype and phenotype” AND “Noonan syndrome” or “RASopathies”, “*PTPN11*” AND “Noonan syndrome” or “RASopathies”, “*SOS1*” AND “Noonan syndrome” or “RASopathies”, “*RAF1*” AND “Noonan syndrome” or “RASopathies”, “*LZTR1*” AND “Noonan syndrome” or “RASopathies”, “*CBL*” AND “Noonan syndrome” or “RASopathies”, “*BRAF*” AND “Noonan syndrome” or “RASopathies”, “*KRAS*” AND “Noonan syndrome” or “RASopathies”, “*SOS2*” AND “Noonan syndrome” or “RASopathies”, and “*RIT1*” AND “Noonan syndrome” or “RASopathies”.

The review of published articles considered the search results and all articles included in their respective bibliographies. Both review studies with extensive case series were selected, and monographs analyzing the genes most rarely involved were considered. Additionally, a temporal criterion was applied, excluding texts published before 2001, the year in which the molecular basis of NS was first described. To obtain a global perspective of the phenotypic expression in NS, no geographic criteria were employed in constructing our database. After removing duplicates, the remaining articles were screened by title and abstract. Preclinical studies, somatic variants, and non-English publications were excluded. The exclusion criteria included incomplete clinical data, absence of genetic testing, inconsistencies in diagnostic definitions, missing key clinical features, and inclusion of patients diagnosed with other syndromes. Studies analyzing genes whose association with NS was not yet fully defined or widely accepted, as well as those reported in single individuals without the potential for statistical analysis, were also excluded.

**Figure 2:**
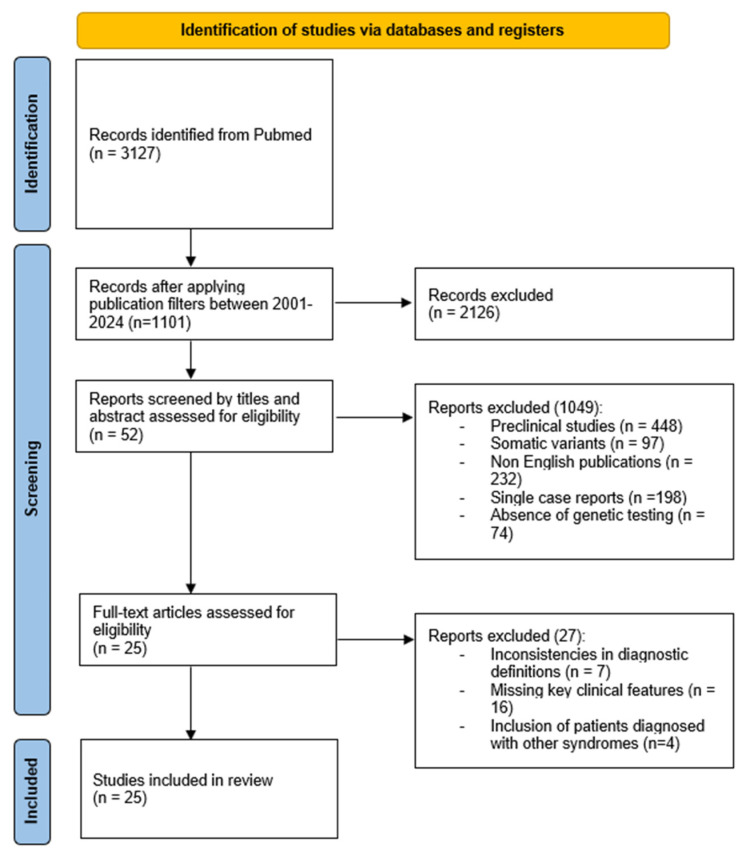
PRISMA flowchart of the study selection process: the results of the PRISM-based process for literature selection illustrate the screening process and article counts resulting from our search. Selection and exclusion criteria are more widely reported in the text of the article.

Following PRISMA guidelines [45], we visually represented the search and selection process. Figure 2 provides a summary of the screening process and the number of articles retrieved based on the search equation and the eligibility criteria applied. From an initial pool of 1101 results, 52 relevant publications were selected based on title and abstract for detailed reading, excluding those with matching titles at the keyword search but differing objectives or methods. Among these, a total of 25 studies were found to be pertinent to the objectives of this research. The selected studies and the corresponding number of analyzed cases are reported in Table 1.

### 2.5. Statistical Analysis

The χ^2^ (or Fisher’s exact when the categories examined had >20% of cells with expected frequencies < 5) test was used to test for differences between genotype groups. A statistical association with *p* < 0.05 was considered significant.

## 3. Results

We recruited a total of 143 patients affected by NS and related disorders (75 males and 68 females). A total of 83.9% of them had Italian origins, 11.2% were North African, 4.2% were European (non-Italian), and 0.7% were South American. Out of them, 116 received a diagnosis of NS, 10 a diagnosis of NSML, 6 a diagnosis of CFCS, 4 of LS, 3 a diagnosis of NS/LAH, and 3 a diagnosis of NSLL.

### 3.1. Genetic Findings

In the 116 patients with a molecularly confirmed clinical diagnosis of NS, pathogenic/likely pathogenic variants were found in *PTPN11* (79, 68.1%), *SOS1* (12, 10.3%), *RAF1* (10, 8.6%), *LZTR1* (either dominant or recessive forms, 9, 7.6%), *BRAF* (2, 1.7%), *KRAS* (2, 1.7%), and *SOS2* and *RIT1* (single case each, 0.9%). Among the investigated families, variants were de novo in 65.7% of cases (46/70) and inherited in 34% (24/70), transmission was maternal in 79% (19/24) of them. A woman with a *PTPN11* variant had identical affected twin daughters, one of whom had an affected child. Two sets of brothers and sisters had *PTPN11* variants; in both cases, one parent was affected but was not included in the study because of the unavailability of extensive data and follow-up. Three of the patients carrying heterozygous *LZTR1* variants were brothers, who inherited the variant from their mother, and the other two brothers with a mutated *LZTR1* allele inherited the variant from their apparently asymptomatic father. In one patient, a girl with pulmonary valve stenosis, short stature, recurrent chylothorax, ptosis, and typical facial dysmorphism, two novel compound heterozygous variants in *LZTR1* (p.Asp668Gly; p.Gln762_Glu765del) were identified. Each variant was inherited by one parent, who showed no NS features, confirming the recessive inheritance model.

CFCS was associated with *MAP2K1* variants in half of the cases (3/6) and with a mutated *BRAF* allele in the other half (3/6). In patients with NSML, variants were found in *PTPN11* (90%, 9/10) and *BRAF* (10%, 1/10); all the variants were de novo, except one with maternal transmission. All three patients with NS/LAH had de novo *SHOC2* variants. LS was confirmed in four patients with *SPRED1* variants: two brothers who inherited the variants from the father, one boy who inherited it from the mother, and one girl with the de novo variant. The three patients with NSLL were brothers, two of them twins, and showed the same *CBL* variant.

### 3.2. Clinical Findings in NS

The clinical characteristics of 116 patients with NS are summarized in Table 2, subgrouped by the involved gene. Overall, 74.1% of patients (86/116) had typical craniofacial features, defined by the evaluating clinician as “mild” in 10.3% of cases (12/116). Short stature (<3°) was observed in 43.1% of individuals (50/116), 26.0% of whom (13/50) required treatment with rGH.

A total of 71.5% of the patients (83/116) had at least one cardiac defect, with 47.0% (39/83) having multiple defects. In 44.6% of cases (37/83), the cardiac complication required surgical correction, and, in 10.8% (9/83), chronic pharmacotherapy was required. The most frequent cardiac finding was pulmonary valve stenosis (PVS) (48.3%, 56/116), followed by an atrial septal defect (ASD) (20.7%, 24/116). An amount of 3.4% of patients (4/116) had arrhythmia, with two of them requiring antiarrhythmic drugs. *SOS1* variants showed a significant association with the presence of multiple cardiopathies (*p* = 0.05). As expected, *PTPN11* variants demonstrated a significant association with PVS and ASD (*p* < 0.05), while those involving *RAF1* were preferentially associated with hypertrophic cardiomyopathy (HCM) (*p* < 0.0001).

Severe neurodevelopmental delay was observed in 24.1% of patients (28/116), associated with cerebral anomalies at MRI in 75.9% of cases. The anomalies observed were highly heterogeneous, reflecting the broad neurological involvement in NS. Among the most frequently reported malformations was the Chiari anomaly. Other findings included delayed myelination, ventriculomegaly, nonspecific white matter abnormalities, and corpus callosum hypoplasia. Some patients also exhibited cortical malformations, such as polymicrogyria, and cerebellar hypoplasia, which could contribute to motor and cognitive impairment. EEG anomalies or epilepsy were reported in 6.0% of cases, requiring treatment in 80% of affected individuals. Epilepsy was associated with *BRAF* variants (*p* = 0.032). Ocular abnormalities were present in 25.0% of cases (29/116) and sensorineural hearing loss in 2.6% (3/116).

Three patients developed juvenile myelomonocytic leukemia (JMML) and one myelodysplasia; all of them carried *PTPN11* variants (*p* = 0.055). One girl died in the first months of life, while the others achieved remission without specific treatments. Three males with *PTPN11* variants developed other malignancies: one subject had testicular juvenile granulosa cell tumor that required surgical excision, one was diagnosed with dysembryoplastic neuroepithelial tumor (DNET) at the age of six during radiological examinations conducted to investigate focal epilepsy (no therapy was performed), and one had a glioma diagnosed at 22 months of life that required specific treatments. One two-year-old girl carrying the *SOS1* variant, presenting with vaginal bleeding, was diagnosed with uterine embryonal pleomorphic rhabdomyosarcoma and, at the time of the article, is undergoing chemotherapy. Genetical and clinical features of individuals with oncological issues are summarized in Table 3.

**Figure 3:**
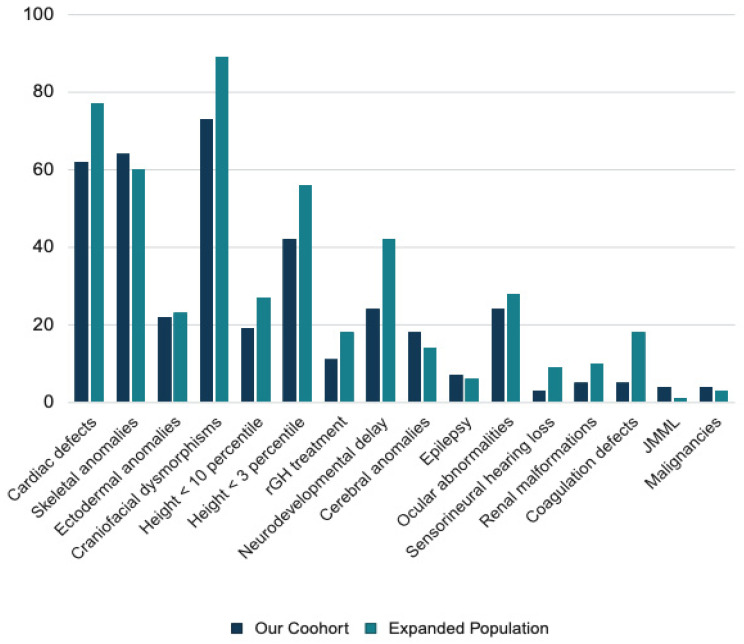
Clinical findings in our population and in the expanded population, expressed as percentages.

The clinical features of 1022 patients with NS worldwide, including our cohort and a further 906 NS cases from the literature review, are summarized in Table 4. Figure 3 illustrates the percentage distribution of clinical features observed in our NS cohort compared to the NS cohort supplemented with cases from the literature. The most common features were cardiac defects (76.2%) and craniofacial dysmorphisms (89.0%). Short stature was common (56.2%) and associated with *KRAS* and *PTPN11* variants. Skeletal anomalies were associated with *PTPN11* variants, while ectodermal anomalies with variants in *SOS1* and *SOS2*. Neurodevelopmental delay, with various degrees of severity, was a common feature in all the studies, variably associated with cerebral anomalies (13.4% of the total population) and epilepsy (5.8%). The prevalence of ocular abnormalities (29.3%) was similar to the one of our population, while hearing loss was more frequent (8.7%). Malignancies occurred in 4.8% and JMML in 1.0% of the affected individuals.

### 3.3. Clinical Finding in Noonan Syndrome with Multiple Lentigines

Individuals with NSML (n = 10) showed typical ectodermal abnormalities in all cases. Short stature was present in 60% (6/10) of cases, mild (between −1.5 and −2 SDS) in 50% of cases, and severe (<−2 SDS) in the other 50%, in one case treated with rGH. Half of the patients had typical craniofacial anomalies. Skeletal anomalies were present in 40% of cases (4/10), visual in 50% (5/10), and auditory in 40% (4/10). In 75% of those cases, implanting hearing aids was necessary. Three patients (30%) had mild heart disease and two (20%) had heart disease requiring surgery, with one female reported to require a heart transplant at 12 months of age. The most frequent cardiac anomaly was HCM (40%, 4/10). Neurodevelopmental delay was present in 50% of patients (5/10) (20% defined as mild and 30% severe), associated with epilepsy in 30%, and with cerebral anomalies in 20% of cases. A total of 30% of patients (3/10) had coagulation disorders. No patient developed malignancies.

Regarding prenatal features, second-trimester ultrasounds documented pathologically increased nuchal translucency in 30% of cases, with a positive triple screen in 20% of cases (2/10).

### 3.4. Clinical Findings in Cardiofaciocutaneous Syndrome

All the patients with CFCS (n = 6) had short stature and craniofacial dysmorphisms. A total of 33% (2/6) of them had prenatal anomalies and 67% (4/6) were born preterm. All patients had cognitive impairment, which was, in 80% of cases, severe, and, in 20%, mild. In 67% of cases (4/6), it was associated with cerebral anomalies at MRI, and, in the same percentage of cases (4/6), with epilepsy. Cardiac abnormalities were present in 50% of the patients (3/6): one patient had an association of PVS, atrial, and ventricular septal defect (VSD), one had persistent left superior vena cava, and one had VSD. A total of 83% of patients (5/6) had visual defects. No malignancies were reported.

### 3.5. Clinical Findings in Legius Syndrome

Common features of patients with LS (n = 4) were the typical ectodermal abnormalities and short stature, which in 50% of cases required rGH therapy, in the absence of severe craniofacial and neurological features. All patients are under auxological and dermatological follow-up. One patient (25%) had ASD and PVS that required surgery at 9 months of age. Prenatal and oncological history was silent.

### 3.6. Clinical Findings in Noonan-like Syndrome with Loose Anagen Hair

Patients with NS/LAH (n = 3) had a relevant prenatal history: in all the cases, second-trimester ultrasounds and triple screens were pathological, and, in two cases (67%), increased nuchal translucency was present. One of the patients (33%) was conceived through medically assisted procreation. All patients had typical craniofacial features, in one case associated with ectodermal abnormalities and in two with short stature (in one case treated with rGH). A total of 67% of patients (2/3) had cerebral anomalies, associated with severe neurodevelopmental delay and, in one case, epilepsy. Cardiovascular manifestations were diverse: one patient had no cardiac abnormalities, one had VSD and mitral valve prolapse for which therapy was not necessary, and one presented with an association of hypertrophic cardiomyopathy, ASD, VSD, and mitral valve dysplasia that needed surgical intervention with closure of the septal defect and placement of a prosthetic valve and a pacemaker and therapy with propafenone and warfarin. None of our patients were diagnosed with malignancies.

### 3.7. Clinical Findings in Noonan Syndrome-like Disorder With or Without Juvenile Myelomonocytic Leukemia

The three patients in our cohort with NSLL were siblings. Genetic evaluation and molecular analysis for Rasopathies were required following the diagnosis of JMML in one of the children, who did not require chemotherapy but was managed with steroid therapy after infectious events. This patient experienced episodes of flare-ups triggered by infectious stimuli, characterized by serositis, lymphadenopathy, splenomegaly, monocytosis, and thrombocytopenia. All three children displayed very subtle dysmorphic features and mild developmental delays; only one (33%) presented with ASD. No information is available about the family of origin to further understand the familial clinical presentation.

## 4. Discussion

The data presented in this study provide valuable insights into the clinical characteristics of different RASopathies, with a particular focus on refining genotype–phenotype correlations. Our primary objective was to comprehensively analyze a large cohort of patients with genetically confirmed Noonan syndrome and related disorders, integrating both clinical and molecular data to enhance our understanding of these conditions. To our knowledge, this is one of the largest single-center studies conducted in Europe on this topic. The extensive sample size enables us to validate many previously reported genotype–phenotype associations in a broader population while also identifying some notable exceptions that warrant further investigation [2,5,6].

### 4.1. Genes Involved in Noonan Syndrome

In patients with NS, the genetic variants identified were predominantly attributable to PTPN11 (68.1%), a proportion higher than the 40–50% typically reported [37,56]. Variants of other genes have been identified in a minority of patients. Variants in SOS1 in the series under examination were found in 10.3% of the patients, a figure lower than the approximate 20% frequency estimated by Roberts et al. [38]. RAF1 variants were found in 9.2% of patients in the series under examination, falling within the known range of 3–17% [39]. The incidence of detected variants of LZTR1, KRAS, BRAF, SOS2, ERF, and RIT1 is consistent with the data reported in the literature [15,21,29,65,66].

### 4.2. Cardiac Phenotype in Noonan Syndrome

Individuals with NS and PTPN11 or SOS1 variants are known to have a high incidence of PS and a lower prevalence of HCM [37,38,65]. In our study, similar results were observed: 48% of patients with PTPN11 variants had PS, while only 10% had HCM. Among patients with SOS1 variants, 90% presented heart disease, with PS being the most prevalent (66%), while HCM was present in 17% of cases.

Our patients with RAF1 variants showed a higher risk of HCM (80%) than the other ones, consistent with previous estimates (87%) [67]. Consequently, strict cardiac follow-up is crucial for patients carrying RAF1 variants.

Finally, in contrast with the literature [68,69], our study did not find a statistically significant association between any cardiac defects and variants in LZTR1 and RIT1.

### 4.3. Growth in Noonan Syndrome

Short stature, defined as height under −2 SD, was present in 43.1% of our cohort with NS and in 56.2% of the cases from the literature review. The average final height in European patients with NS is approximately 152.7 cm in women and 162.5 cm in men [70]. While normal final height is observed in 30% of individuals with NS, over 50% of women and nearly 40% of men have a height below the third percentile [71,72]. The genetic basis of poor growth and short stature in NS is not fully understood. The hyperactivation of the RAS-MAPK pathway is likely involved in both altered growth hormone (GH) secretion and reduced peripheral response to GH [73]. The association between PTPN11 and short stature, which is statistically significant in our study, is well known and documented in the literature [74,75]. Additionally, it has been suggested that PTPN11 variants may be negatively correlated with response to rGH therapy [76]. In contrast, until now, an association between KRAS and short stature in Noonan syndrome has not been reported. This study highlights an association; although previous studies have documented sporadic cases, the limited time since KRAS was identified in relation to NS has hindered the establishment of a sufficiently large cohort to achieve statistically significant results. The use of rGH therapy is currently under discussion, although it has been authorized by regulatory agencies in several countries, including Italy, since 2021 [4,77]. Currently, it is established that rGH treatment is effective in increasing growth velocity in the short to medium term and some studies have also shown an improvement in final height in NS [73,78,79,80,81]. Overall, 11.2% of our patients received treatment with rGH, but the advancing understanding of the genetic basis of short stature in NS and the implementation of new regulations will make it possible to offer this treatment to a greater number of individuals. Furthermore, feeding problems and failure to thrive during the early years of life may also contribute to the final short stature observed in NS patients [82]. The importance of adequate nutrition during these critical stages of development cannot be overstated, as early nutritional deficiencies can significantly impact growth and overall development. These factors, in combination with genetic influences, likely play a role in determining final stature.

### 4.4. Cancer Risk in Noonan Syndrome

In the current analysis of the clinical aspects of NS, a central topic of debate is the increased oncological risk and the potential need for tumor surveillance. The RAS/MAPK signaling pathway plays a pivotal role in somatic tumor development [83]; however, oncological risk does not appear significantly elevated in older children and adults with NS compared to the general population [84]. In younger affected children, however, the cancer risk is estimated to be approximately eight times higher than in children without NS [84]. This higher prevalence in early childhood is primarily attributable to JMML, a rare myeloproliferative disorder. It is important to note that in most cases, affected children exhibit a JMML-like myeloproliferative condition that does not require treatment and typically undergoes spontaneous resolution [85,86]. In our study population, this condition presented within the first few months of life and, in most cases, remained stable without therapeutic intervention. The association with JMML is particularly significant in individuals with PTPN11 variants, as confirmed in our study [84,86]. However, the precise molecular mechanisms underlying why children with Noonan syndrome are more prone to developing JMML remain unclear. This is an important area of research for the future, as understanding the genetic and molecular basis of this predisposition could provide valuable insights into potential preventive or therapeutic strategies.

Updated recommendations from the American Association for Cancer Research emphasize that routine complete blood count (CBC) surveillance in otherwise healthy children with NS is not warranted, aligning with guidelines for other cancer predisposition syndromes [87,88]. Instead, close clinical monitoring, particularly in infancy and early childhood, is recommended, with attention to hepatosplenomegaly and other early signs of JMML. For individuals with high-risk variants (such as PTPN11 p.T73I and KRAS p.T58I), oncological screening with physical examination and CBC every 3–6 months until five years of age is advised.

Regarding solid tumors, a potential association between NS and DNET has been described in previous case reports, and the inclusion of our case in this cohort provides additional evidence supporting this hypothesis [89]. A similar concern exists for rhabdomyosarcoma [90] and for gliomas and glioneuronal tumors [91]. However, current data on the overall risk of solid tumors in NS remain insufficient [88]. Notably, recent recommendations do not contraindicate the use of growth hormone (GH) therapy in NS due to a lack of evidence supporting an increased tumor risk associated with its administration [87]. In our cohort, the overall percentage of individuals with malignancies was 7.7%. This figure may represent an underestimation, partly due to the absence of long-term follow-up into adulthood in some cases, and warrants further investigation.

### 4.5. Neurodevelopmental Phenotype in Noonan Syndrome

In our cohort of patients diagnosed with NS, epilepsy was predominantly observed in association with neurodevelopmental delay, and its overall incidence was determined to be 6.0%. This figure surpassed that of the general population, which is estimated to range from 0.6 to 1% [92,93]. Likewise, the prevalence of cerebral anomalies among our NS patients was notably higher at 17.2% compared to the general population rate of 9.8 per 10,000 individuals [94]. The RAS/MAPK pathway is involved in the organization of the central nervous system (CNS) both functionally and structurally: it is implicated in controlling cell division and differentiation during development, dendritic organization in differentiated neurons, and the promotion of synaptic connectivity among cortical neurons [95]. The broad role of the RAS/MAPK pathway in multiple aspects of brain development is reflected in the wide variability of cerebral malformations observed in NS, ranging from Chiari malformation and ventriculomegaly to white matter abnormalities and cortical dysplasias. While epilepsy has been previously associated with RASopathies [96,97,98], there has been no previous comprehensive study reporting a high incidence of structural malformations of the CNS and spinal cord in those conditions. Our findings are likely attributed to the increased availability of radiological examinations and the fact that our center serves as a referral center where children with complex pathologies are sent for neurological evaluations. Given the heterogeneity in the presentation of neurodevelopmental disorders within RASopathies and the challenges in diagnosing conditions such as autism spectrum disorder (ASD) or attention deficit hyperactivity disorder (ADHD) at younger ages, we focused our assessment on neurodevelopmental delay and milestone achievement. This approach, while standardizing the assessment, may represent a limitation in fully capturing the breadth of neurodevelopmental comorbidities associated with NS. Emerging studies have indeed highlighted a higher prevalence and severity of autism traits in NS and other RASopathies [99]. The results of our study emphasize the importance of comprehensive neurological evaluations for RASopathy patients, extending beyond assessments of neurodevelopmental and cognitive aspects, and encompassing the examination of additional neurological manifestations and, when necessary, radiological imaging.

### 4.6. Other Clinical Features of Noonan Syndrome

Ocular anomalies are a frequent feature of NS, observed in 29.3% of cases. The prevalence was higher in individuals with RIT1 (70.7%) and SOS1 (40.0%) variants compared to those with PTPN11 (20.1%). These anomalies include refractive errors, ptosis, and strabismus [2]. Ophthalmological screening could be recommended at diagnosis, particularly for patients with RIT1 variants, with follow-up tailored based on initial findings.

Skeletal anomalies are observed in approximately 60% of patients with NS, with a statistically significant association with PTPN11 variants. Common findings include chest deformities, scoliosis, and joint hypermobility [5,37]. Clinicians managing patients with NS should always include regular assessments of spinal curvature and other key skeletal features during follow-up visits to detect abnormalities early and refer them to an orthopedic specialist when necessary.

### 4.7. Molecular Correlations in Noonan Syndrome

NS is fundamentally a disorder of disrupted signaling within the RAS/MAPK pathway, a critical cascade for cellular processes such as proliferation, differentiation, and survival.

Variants in PTPN11, the most frequently affected gene in NS (68.1% of cases in our cohort), result in a gain of function of SHP2, a tyrosine phosphatase. As demonstrated in murine models, this aberrant activation enhances downstream ERK phosphorylation, which has been implicated in the abnormal development of semilunar cardiac valves, explaining the high prevalence of PVS in PTPN11-positive patients [100]. Additionally, PTPN11 variants have been implicated in growth impairment through their effect on GH signaling. NS models with SHP2 gain-of-function variants exhibit reduced IGF-1 levels due to RAS/ERK1/2 hyperactivation, which disrupts normal GH-induced IGF-1 release [73].

*SOS1* variants (10.3% of cases) similarly upregulate the RAS/MAPK pathway but through enhanced guanine nucleotide exchange factor (GEF) activity, which accelerates RAS activation. This hyperactivation explains the predominance of multiple cardiac defects, including PVS and ASD [38,101]. Notably, SOS1 variants have also been associated with ectodermal anomalies, likely due to the pathway’s role in skin and hair development [60].

In contrast, variants in RAF1 (8.6% of cases) result in hyperactive kinase activity, driving excessive ERK phosphorylation. This molecular dysregulation underpins the striking association of RAF1 with HCM, observed in 80% of cases in our cohort. The hypertrophy is thought to arise from abnormal cardiomyocyte proliferation and survival, consistent with observations in animal models of RAF1 overexpression [102].

*KRAS* variants, though less frequent, act upstream in the pathway and have a particularly potent effect on RAS activation. This may explain the more severe phenotypes observed in KRAS-positive individuals, including profound growth failure and neurodevelopmental delay [54,66].

*LZTR1*, a gene recently associated with autosomal recessive NS, acts as a negative regulator of RAS signaling through the ubiquitination of RAS proteins [103]. Loss-of-function variants in LZTR1 likely remove a critical checkpoint in this pathway, contributing to a spectrum of features including mild dysmorphisms and cardiac anomalies, although with a reduced prevalence of hypertrophic cardiomyopathy compared to RAF1 or PTPN11 [36,40].

*RIT1*, identified in approximately 5–10% of NS cases, encodes a small GTPase that modulates oxidative stress responses and cell proliferation. Gain-of-function variants lead to hyperactivation of downstream signaling, often resulting in a high prevalence of cardiac anomalies such as PVS and atrial defects, alongside characteristic facial dysmorphisms and an increased incidence of prenatal abnormalities like polyhydramnios [22,69].

*SOS2*, a homolog of SOS1, has been implicated in only a small number of NS cases. Similarly to SOS1, its variants enhance RAS activation, with preliminary evidence suggesting overlapping clinical features such as ectodermal anomalies and cardiac defects. However, the limited number of reported cases precludes robust genotype–phenotype correlations [14,21].

Lastly, BRAF variants, primarily associated with CFCS, are occasionally identified in NS. These variants lead to constitutive kinase activation, which enhances ERK signaling. Patients with BRAF variants often exhibit a high prevalence of ectodermal anomalies, neurodevelopmental delay, and cardiac defects, including hypertrophic cardiomyopathy [29,104].

The detailed molecular understanding of these variants underscores the heterogeneity of NS. Each gene contributes uniquely to the disruption of the RAS/MAPK pathway, producing distinct yet overlapping clinical phenotypes.

### 4.8. Other RASopathies

All subjects with NSML carried heterozygous variants in *PTPN11*, as expected [33,105]. Despite the frequency being lower than the previously reported 70% [34,105], HCM remained the most prevalent cardiac abnormality. In our population, the high incidence of hearing impairment is confirmed to be 40% [105,106].

In our center cohort, CFCS was equally associated with *MAP2K1* and *BRAF* variants (60%), similar to the literature, where 60% of the cases are attributed to *BRAF* variants [5,30]. The phenotype was typically severe, with neurodevelopmental delay, mostly associated with epilepsy and cerebral anomalies. Craniofacial and cutaneous features are typical and similar to the ones of patients from other works [5,30,107].

NS/LAH is the less frequent RASopathy. In all cases, as we confirm, it is associated with *SHOC2* variants [19]. Growth deficit and valvular and septal defects are common features, with higher reported prevalence than in the NS [19,108]. However, due to the small number of patients in our study, we cannot infer definitive conclusions regarding these associations.

As expected, LS had a milder phenotype than the other RASopathies, with short stature being the mean feature together with dermatological anomalies [5,6]. It is fundamental to provide these patients with regular auxological follow-up to enable early initiation of appropriate treatment, such as rGH, if necessary.

NSLL is one of the last RASopathies identified and is classically associated with *CBL* variants. This gene is still being studied; although *CBL* was first linked to a Noonan-like syndrome by Martinelli et al. [17], it has only recently been recognized as part of the NS clinical spectrum [16,109]. The increased risk of JMML is characteristic and a useful diagnostic handle for this condition, as demonstrated by our affected patient.

## 5. Conclusions

Phenotypic differences in RASopathies arise from the distinct biological roles of the affected genes within the RAS/MAPK pathway, the degree of pathway dysregulation induced by specific pathogenic variants, and the tissue-specific impact of altered signaling. Variants in different genes can lead to varying levels of pathway activation, influencing the severity of clinical manifestations. Additionally, the hierarchical position of each protein within the pathway and its interaction with other signaling molecules contribute to tissue-specific effects, explaining the variability in features such as cardiac defects, neurodevelopmental outcomes, and ectodermal anomalies. These differences reflect the complex interplay of genotype, pathway dynamics, and developmental timing, underscoring the importance of understanding these mechanisms to improve diagnosis, prognosis, and targeted therapeutic strategies.

This study represents the largest genotype–phenotype analysis with a review of the literature conducted to date on NS and other RASopathies. By integrating data from a large cohort of patients and combining both previously reported and newly described cases from diverse regions worldwide, this work underscores the value of consolidating global evidence to refine our understanding of RASopathies. It highlights the importance of exploring genotype–phenotype correlations, particularly for less common genes, to better delineate their clinical implications. Continuous research into these correlations, alongside newly generated data, will further enhance precision in patient management.

Future research directions should focus on defining how the same pathogenic variant can lead to different phenotypic outcomes and identifying additional genetic or environmental modifiers influencing RASopathy manifestations. A better understanding of the molecular mechanisms involved could pave the way for novel therapeutic approaches, including targeted treatments for hypertrophic cardiomyopathy and other complications. Furthermore, research is needed to assess the long-term efficacy and safety of growth hormone therapy in these patients and to evaluate the lifelong quality of life in individuals with RASopathies. Expanding our knowledge in these areas will be critical for optimizing patient care and developing new treatment strategies.

## Figures and Tables

**Table 1:** List of the selected articles.

Title	DOI	Reference	Number of Cases
Phenotype–genotype analysis of 242 individuals with RASopathies: 18-year experience of a tertiary center in Brazil	DOI: 10.1002/ajmg.c.31851	[46]	181
**This cohort**			116
Molecular and clinical studies in 107 Noonan syndrome affected individuals with *PTPN11* variants	DOI: 10.1186/s12881-020-0986-5	[47]	107
Molecular and phenotypic spectrum of Noonan syndrome in Chinese patients	DOI: 10.1111/cge.13588	[48]	102
Genotypic and Phenotypic Characterization of Noonan Syndrome: New Data and Review of the Literature	DOI: 10.1002/ajmg.a.30598	[49]	56
*PTPN11* Variants in Noonan Syndrome: Molecular Spectrum, Genotype–Phenotype Correlation, and Phenotypic Heterogeneity	DOI: 10.1086/340847	[37]	54
Spectrum of variants and genotype–phenotype analysis in Noonan syndrome patients with *RIT1* variants	DOI: 10.1007/s00439-015-1627-5	[23]	48
Genotype–phenotype correlation analysis in Japanese patients with Noonan syndrome	DOI: 10.1507/endocrj.EJ18-0564	[50]	39
Genotype–phenotype correlations in Noonan syndrome	DOI: 10.1016/j.jpeds.2003.11.032	[51]	34
Genotype and phenotype in patients with Noonan syndrome and a RIT1 mutation	DOI: 10.1038/gim.2016.32	[52]	33
Spectrum of variants in *PTPN11* and genotype–phenotype correlation in 96 patients with Noonan syndrome and five patients with cardio-facio-cutaneous syndrome	DOI: 10.1038/sj.ejhg.5200935	[53]	32
*PTPN11*, *SOS1*, *KRAS*, and *RAF1* gene analysis, and genotype–phenotype correlation in Korean patients with Noonan syndrome	DOI: 10.1007/s10038-008-0343-6	[54]	30
Molecular and clinical profile of patients referred as Noonan or Noonan-like syndrome in Greece: a cohort of 86 patients	DOI: 10.1007/s00431-022-04574-w	[55]	28
Correlation between *PTPN11* gene variants and congenital heart defects in Noonan and LEOPARD syndromes	DOI: 10.1136/jmg.40.9.704	[56]	23
*PTPN11* Gene Analysis in 74 Brazilian Patients with Noonan Syndrome or Noonan-like Phenotype	DOI: 10.1089/gte.2006.10.186	[57]	21
Mutational analysis of the *PTPN11* gene in Egyptian patients with Noonan syndrome	DOI: 10.1016/j.jfma.2012.06.002	[58]	21
Mutation and Phenotypic Spectrum of Patients With RASopathies	PMID: 33452774	[59]	19
Rare variants in *SOS2* and *LZTR1* are associated with Noonan syndrome	DOI: 10.1136/jmedgenet-2015-103018	[21]	18
Clinical and molecular analysis of RASopathies in a group of Turkish patients	DOI: 10.1111/j.1399-0004.2012.01875.x	[60]	15
Providing more evidence on *LZTR1* variants in Noonan syndrome patients	DOI: 10.1002/ajmg.a.61445	[36]	14
Clinical and Molecular Findings of Tunisian Patients with RASopathies	DOI: 10.1159/000362898	[61]	11
Variable phenotypic expression in a large Noonan syndrome family segregating a novel *SOS1* mutation	DOI: 10.1002/ajmg.a.38466	[62]	10
Delineation of dominant and recessive forms of *LZTR1*-associated Noonan syndrome	DOI: 10.1111/cge.13533	[63]	9
Novel variants and their genotype–phenotype correlations in patients with Noonan syndrome, using next-generation sequencing	DOI: 10.1016/j.advms.2017.07.001	[64]	8
Activating Variants Affecting the Dbl Homology Domain of *SOS2* Cause Noonan Syndrome	DOI: 10.1002/humu.22834	[14]	7
Noonan Syndrome in South Africa: Clinical and Molecular Profiles	DOI: 10.3389/fgene.2019.00333	[44]	3

**Table 2:** Clinical findings in our population with Noonan syndrome (NS), according to the involved genes.

	*PTPN11* (*n* = 79)	*SOS1* (*n* = 12)	*RAF1* (*n* = 10)	*LZTR1* (*n* = 9)	*BRAF* (*n* = 2)	*KRAS* (*n* = 2)	*RIT1* (*n* = 1)	*SOS2* (*n* = 1)	Total (*n* = 116)
***Cardiac features***									
***Cardiopathy***									
Combination	28 (35.4%)	7 (58.3%)	4 (40.0%)	-	-	-	-	-	39 (33.6%)
PVS	37 (46.8%)	8 (66.7%)	4 (40.0%)	3 (33.3%)	2 (100%)	1 (50.0%)	1 (100%)	-	56 (48.3%)
ASD	23 (19.0%)	1 (8.3%)	-	-	-	-	-	-	24 (20.7%)
VSD	1 (1.3%)	4 (33.3%)	-	-	-	-	-	1 (100%)	6 (5.2%)
HCM	7 (8.9%)	2 (16.7%)	8 (80.0%)	-	-	-	-	-	17 (14.7%)
AS	4 (5.1%)	-	1 (10.0%)	-	-	-	-	-	5 (4.3%)
AVD	3 (3.8%)	1 (8.3%)	-	-	-	-	-	-	4 (3.4%)
MVD	6 (7.6%)	2 (16.7%)	2 (20.0%)	-	-	-	-	-	10 (8.6%)
TVD	1 (1.3%)	-	1 (10.0%)	-	-	-	-	-	2 (1.6%)
TOF	1 (1.3%)	-	-	-	-	-	-	-	1 (0.9%)
AVC	2 (2.5%)	-	-	-	-	-	-	-	2 (1.7%)
PVRA	1 (1.3%)	-	-	-	-	-	-	-	1 (0.9%)
PDA	-	3 (25.0%)	-	-	-	-	-	-	3 (2.6%)
None	23 (29.1%)	1 (8.3%)	2 (20.0%)	6 (66.7%)	-	1 (50.0%)	-	-	33 (28.4%)
***Severity of cardiac phenotype***									
Mild cardiopathy	11 (13.9%)	7 (58.3%)	2 (20.0%)	3 (33.3%)	1 (50.0%)	1 (50.0%)	-	1 (100%)	26 (22.4%)
Cardiopathy requiring chronic drug therapy	4 (5.1%)	1 (8.3%)	4 (40.0%)	-	-	-	-	-	9 (7.8%)
Cardiopathy requiring surgery	28 (35.4%)	4 (33.3%)	1 (10.0%)	1 (11.1%)	1 (50.0%)	1 (50.0%)	1 (100%)	-	37 (31.9%)
Severe cardiopathy with death	-	-	1 (10.0%)	-	-	-	-	-	1 (0.9%)
Arrhythmia	2 (2.5%)	-	2 (20.0%)	-	-	-	-	-	4 (3.4%)
***Orthopedic features***									
Skeletal anomalies	33 (41.8%)	7 (58.3%)	2 (20.0%)	3 (33.3%)	1 (50.0%)	2 (100%)	1 (100%)	1 (100%)	50 (43.1%)
Severe scoliosis	4 (5.1%)	1 (8.3%)	-	-	-	-	-	-	5 (4.3%)
Severe pectus excavatus	-	1 (8.3%)	-	-	-	-	-	-	1 (0.9%)
***Ectodermal features***									
Ectodermal anomalies	8 (10.1%)	3 (25.0%)	4 (40.0%)	1 (11.1%)	1 (50.0%)	1 (50.0%)	-	1 (100%)	19 (16.4%)
Severe craniofacial dysmorphisms	45 (56.9%)	11 (91.7%)	9 (90.0%)	5 (55.5%)	1 (50.0%)	2 (100%)	-	1 (100%)	74 (63.8%)
Mild craniofacial dysmorphisms	7 (8.9%)	-	-	3 (33.3%)	1 (50.0%)	-	1 (100%)	-	12 (10.3%)
***Prenatal features***									
Medically assisted reproduction	5 (6.3%)	1 (8.3%)	-	1 (11.1%)	-	1 (50.0%)	U	-	8 (6.9%)
Intrauterine growth restriction	7 (8.9%)	-	1 (10.0%)	-	-	-	U	-	8 (6.9%)
Polyhydramnios	20 (25.3%)	6 (50.0%)	5 (50.0%)	1 (11.1%)	1 (50.0%)	-	U	1 (100%)	34 (29.3%)
Oligohydramnios	2 (2.5%)	-	-	-	-	-	U	-	2 (1.7%)
Pathological second-trimester ultrasound	30 (37.9%)	7 (58.3%)	5 (50.0%)	1 (11.1%)	2 (100.0%)	-	U	1 (100%)	46 (39.6%)
Increased nuchal translucency	8 (10.1%)	6 (50.0%)	-	1 (11.1%)	-	-	U	-	15 (12.9%)
Positive triple screen	9 (11.4%)	2 (16.7%)	-	-	-	-	U	-	11 (9.5%)
Twin pregnancy	1 (1.3%)	-	-	1 (11.1%)	-	-	U	-	2 (1.7%)
Premature birth	12 (15.2%)	7 (58.3%)	5 (50.0%)	1 (11.1%)	1 (50.0%)	2 (100%)	U	-	28 (24.1%)
***Growth***									
Height < 10°	19 (24.1%)	1 (8.3%)	-	2 (22.2%)	-	-	-	-	22 (19.0%)
Height < 3°	36 (45.6%)	4 (33.3%)	4 (40.0%)	2 (22.2%)	2 (100.0%)	2 (100%)	-	-	50 (43.1%)
rGH treatment	13 (16.5%)	-	-	-	-	-	-	-	13 (11.2%)
***Neurological features***									
Severe neurodevelopmental delay	16 (20.3%)	5 (41.7%)	2 (20.0%)	2 (22.2%)	2 (50.0%)	-	-	1 (100%)	28 (24.1%)
Cerebral anomalies at MRI	13 (16.5%)	2 (16.7%)	3 (30.0%)	-	1 (50.0%)	-	1 (100%)	-	20 (17.2%)
Epilepsy	3 (3.8%)	1 (8.3%)	1 (10.0%)	-	1 (50.0%)	1 (50.0%)	-	-	7 (6.0%)
Ocular abnormalities	18 (22.8%)	6 (50.0%)	2 (20.0%)	1 (11.1%)	1 (50.0%)	1 (50.0%)	-	-	29 (25.0%)
Sensorineural hearing loss	2 (2.5%)	-	1 (10.0%)	-	-	-	-	-	3 (2.6%)
***Urological features***									
Cryptorchidism	26/39 (66.7%)	6/8 (75.0%)	1/4 (25.0%)	2/7 (28.6%)	0/2 (0.0%)	0/0 (0.0%)	1/1 (100%)	1/1 (100%)	37/62 (59.7%)
Renal malformations	4 (5.1%)	1 (8.3%)	2 (20.0%)	-	-	-	-	-	7 (6.0%)
***Hematological features***									
Coagulation defects	2 (2.5%)	2 (16.7%)	1 (10.0%)	-	-	1 (50.0%)	-	-	6 (5.2%)
JMML	4 (5.1%)	-	-	-	-	-	-	-	4 (3.5%)
Other malignancies	4 (5.1%)	1 (8.3%)	-	-	-	-	-	-	5 (4.3%)

PVS: pulmonary valve stenosis; ASD: atrial septal defect; VSD: ventricular septal defect; HCM: hypertrophic cardiomyopathy; AS: aortic stenosis; AVD: aortic valve dysplasia; MVD: mitral valve dysplasia; TVD: tricuspid valve dysplasia; TOF: Tetralogy of Fallot; AVC: atrioventricular canal; PVRA: pulmonary venous return anomaly; PDA: patent ductus arteriosus; U: unknown; rGH: recombinant growth hormone; MRI: magnetic resonance imaging; JMML: juvenile myelomonocytic leukemia.

**Table 3:** Clinical and genetic features of our patients with RASopathies and oncological complications.

Sex	Gene	Coding Variant NM_002834.5	Protein Variant	Inherited	Diagnosis	Age at Diagnosis	Treatment	Other Relevant Clinical Features	Death
**M**	*PTPN11*	c.417G>C	p.(Glu139Asp)	No	**Myelodysplasia**	9 months	No	PS, ASD, typical craniofacial dysmorphisms	No
**M**	*PTPN11*	c.561G>A	p.(Asp61Asn)	No	**JMML**	At birth	No	Dorsal mast cell tumor, AVC, vWF deficiency, cryptorchidism, short stature, mild neurodevelopmental delay	No
**M**	*PTPN11*	c.797G>C	p.(Glu139Asp)	No	**JMML**	3 months	No	Cerebral palsy, ASD, aortic ectasia, cerebral hamartomas	No
**F**	*PTPN11*	c.845T>G	p.(Phe285Ile)	No	**JMML**	At birth	Corticosteroids	HCM, PS, severe lymphatic dysplasia, typical craniofacial dysmorphisms	Yes at 21 months
**F**	*CBL*	c.1222T>C	p.(Trp408Arg)	Yes (M)	**JMML**	25 months	Corticosteroids during flares	Recurrent vasculitis, mild craniofacial dysmorphisms, mild neurodevelopmental delay	No
**M**	*PTPN11*	c.1851C>A	p.(Pro491His)	No	**DNET**	72 months	No	VSD, ectopic atrial tachycardia, focal epilepsy	No
**M**	*PTPN11*	c.241G>T	p.(Ala72Ser)	No	**Glioma**	22 months	Surgical exeresis, trametinib, dabrafenib	ASD, mild craniofacial dysmorphisms, mild neurodevelopmental delay	No
**M**	*PTPN11*	c.794G>A	p.(Arg265Gln)	Yes (M)	**Testicular juvenile granulosa cell tumor**	12 months	Orchidectomy	Mild craniofacial dysmorphisms	No
**F**	*SOS1*	c.797c>A	p.(Thr266Lys)	No	**Embryonal pleomorphic rhabdomyosarcoma**	24 months	Chemotherapy †	ASD, AVC, typical craniofacial dysmorphisms	No

JMML: juvenile myelomonocytic leukemia; PS: pulmonary stenosis; ASD: atrial septal defect; AVC: atrioventricular canal; vWF: von Willebrand factor; HCM: hypertrophic cardiomyopathy; DNET: dysembryoplastic neuroepithelial tumor; VSD: ventricular septal defect, M: maternal inheritance; † ongoing treatment.

**Table 4:** Clinical findings in the expanded population with Noonan syndrome (NS), according to gene variants.

	*PTPN11* (*n* = 675)	*SOS1* (*n* = 87)	*RAF1* (*n* = 42)	*LZTR1* (*n* = 59)	*BRAF* (*n* = 9)	*KRAS* (*n* = 30)	*RIT1* (n = 102)	*SOS2* (*n* = 18)	Total (*n* = 1022)	ANOVA *p* Value
Cardiac defects	517/665 (77.7%)	57/84 (67.8%)	40/42 (95.2%) *	32/59 (54.2%)	8/9 (88.9%)	26/29 (89.6%)	88/102 (86.3%)	10/18 (55.5%)	778/1021 (76.2%)	<0.0001
Skeletal anomalies	377/555 (67.9%) *	38/79 (48.1%)	17/41 (41.5%)	32/59 (54.2%)	4/7 (57.1%)	11/27 (40.7%)	45/89 (50.6%)	9/18 (50.0%)	533/875 (60.9%)	<0.0001
Ectodermal anomalies	41/294 (13.9%)	20/48 (41.7%) *	7/30 (23.3%)	8/43 (18.6%)	2/6 (33.3%)	4/10 (40%)	28/71 (39.4%)	6/8 (75.0%) *	116/510 (22.7%)	<0.0001
Craniofacial dysmorphisms	507/581 (87.6%)	75/80 (93.7%)	35/36 (97.2%)	57/59 (96.6%)	7/7 (100%)	22/25 (88.0%)	46/55 (83.6%)	11/11 (100%)	760/854 (89.0%)	0.0422
Height < 10°	93/251 (37.1%) *	7/40 (17.5%)	1/23 (4.3%)	6/36 (16.7%)	0/6 (0%)	0/10 (0%)	4/36 (11.1%)	2/13 (15.4%)	113/415 (27.2%)	<0.0001
Height < 3°	376/591 (63.6%) *	18/80 (22.5%)	26/41 (63.4%)	9/49 (18.4%)	5/7 (71.4%)	20/27 (74.1%) *	34/65 (52.3%)	4/15 (26.7%)	492/875 (56.2%)	<0.0001
rGH treatment	39/162 (24.1%)	2/30 (6.7%)	3/23 (13.0%)	1/22 (4.5%)	1/4 (25.0%)	0/9 (0%)	0/9 (0%)	0/10 (0%)	46/269 (17.1%)	0.0281
Neurodevelopmental delay	194/499 (38.9%)	32/73 (43.8%)	13/33 (39.4%)	23/50 (46.0%)	5/7 (71.4%)	18/25 (72.0%) *	26/49 (53.1%)	7/16 (43.7%)	318/752 (42.3%)	0.0241
Cerebral anomalies at MRI	17/190 (8.9%)	4/27 (14.8%)	4/22 (18.2%)	2/29 (6.9%)	2/7 (28.6%)	2/12 (16.7%)	10/10 (100%) *	0/9 (0%)	41/306 (13.4%)	<0.0001
Epilepsy	12/207 (5.7%)	2/33 (6.0%)	2/21 (9.5%)	0/26 (0%)	3/7 (42.9%) *	1/12 (8.3%)	2/45 (4.4%)	0/14 (0%)	22/376 (5.8%)	0.0007
Ocular abnormalities	31/154 (20.1%)	16/40 (40.0%)	3/18 (16.7%)	7/46 (15.2%)	1/2 (50.0%)	5/12 (41.7%)	29/41 (70.7%) *	2/8 (25.0%)	94/321 (29.3%)	<0.0001
Sensorineural hearing loss	21/199 (10.5%)	4/57 (7.0%)	3/41 (7.3%)	1/19 (5.3%)	0/6 (0%)	2/18 (11.1%)	3/43 (6.9%)	0/8 (0%)	34/391 (8.7%)	0.9347
Cryptorchidism	185/311 (59.5%)	23/48 (47.9%)	5/23 (16.1%)	5/17 (29.4%)	1/4 (25.0%)	1/15 (6.7%)	25/40 (62.5%)	7/8 (87.5%)	252/466 (54.1%)	<0.0001
Renal malformations	22/266 (8.2%)	7/50 (14.0%)	5/31 (16.1%)	2/51 (3.9%)	0/5 (0%)	2/20 (10%)	10/44 (22.7%)	2/17 (11.8%)	50/484 (10.3%)	0.0936
Coagulation defects	66/352 (18.7%)	14/52 (26.9%)	7/36 (19.4%)	5/53 (9.4%)	0/5 (0%)	3/22 (13.6%)	12/51 (23.5%)	6/17 (35.3%)	113/588 (19.2%)	0.0919
JMML	6/376 (1.6%)	0/56 (0%)	0/36 (0%)	0/39 (0%)	0/9 (0%)	0/24 (0%)	0/22 (0%)	0/17 (0%)	6/579 (1.0%)	0.4077
Malignancies	17/398 (4.3%)	5/65 (7.7%)	2/42 (4.8%)	2/53 (3.8%)	0/9 (0%)	1/30 (3.3%)	5/54 (9.3%)	0/9 (0%)	32/660 (4.8%)	0.6791

Percentages refer to the number of patients for whom the specific clinical information was available. * indicates the gene for which a statistically significant correlation with the phenotype has been established.

## Data Availability

The original contributions presented in this study are included in the article. Further inquiries can be directed to the corresponding author.
